# Supplementary material for: Normative Data for Learning and Memory Test (TAMV-I) in Latin American and Spanish Children: An item response theory and linear mixed models approach
Source: PLoS One. 2026 Feb 18;21(2):e0341237. doi: 10.1371/journal.pone.0341237 (PMC12915919; doi:10.1371/journal.pone.0341237)
Supplement: Attachment — Submitted filename: Response to Reviewers.docx [file pone.0341237.s003.docx]

**Reviewer #1**

Overall, the study addresses an important issue in neuropsychological testing: the lack of access to standardized and high-quality neuropsychological assessments and the limitations of traditional statistical methods (e.g., Classical Test Theory, CTT) in generating reliable normative data. However, the discussion regarding country-specific effects and the variability in normative data interpretation remains underdeveloped. The study raises a critical question: If performance varies significantly across demographic factors, how should normative data be applied effectively in diverse populations?

**Response:** Thank you for your incisive comment. We think you have touched on a central issue we attempt to address in this study. Performance on neuropsychological measures does vary significantly across demographic factors, which presents an important challenge when attempting to develop representative norms. Traditionally, norms have been stratified by age groups and, less often, by other variables such as education or ethnicity/race. However, this approach suffers from two limitations: 1) we do not know a priori which variables are related to neuropsychological performance; and, 2) stratifying norms reduces power by limiting the amount of subjects in each category. In order to address these and other issues, we chose to use regression-based norming in this study. By doing so we can identify which variables are affecting performance beforehand and include them in the regression equation, which can transform direct scores to standardized values that account for demographic variables. Given that this approach identifies variables that affect performance, this variability is controlled for in the regression model, thus providing accurate norms for several demographic groups, including age, gender, education, and country related groupings. We added a brief explanation of this issue in the discussion, lines 470-473.

The presentation of results is unclear, making it challenging to extract key findings.

**Response:** We appreciate this observation. To improve the clarity and accessibility of the results, we have restructured the Methods and Results sections.

Figures should be more structured and detailed, with improved resolution for readability. Add notes explaining the main features of figures.

**Response:** Thank you for this suggestion. We have reformatted all figures to enhance readability by increasing resolution, enlarging font sizes, and improving legends. In addition, we have added explanatory notes on the figures to highlight their main features and guide interpretation (see line 479).

Sampling Procedure: It is not explicitly stated how participants were selected.

**Response:** We thank the reviewer for this observation. In the revised Procedure section (page 8), we have clarified the sampling process. Local research teams in each country first established agreements with schools and high schools to present the study. After obtaining authorization from the institutions, students and their families were informed about the project and invited to participate voluntarily.

Sampling Procedure: Was the sample size for each country predetermined, or was it subject to availability?

**Response:** The sample size for each country was subject to availability at the collaborating institutions rather than predetermined. However, local research teams ensured balanced distributions across sex and age groups, and mean parental education (MPE) was monitored in each subsample (see Table 1). This approach enhanced representativeness despite the convenience sampling strategy. Moreover, the final sample size allowed the calculation of sampling error, supporting the reliability of the normative estimates. Following Innocenti et al. (2023), and assuming a 95% confidence level, and with z₀ = –0.954, the expected standard error was 0.2679 for Spain, 0.3153 for Honduras, 0.2503 for Colombia, 0.2178 for Ecuador, and 0.1280 for the total sample. These values fall within acceptable ranges, confirming that the achieved precision is sufficient for the study aims and for the generation of robust and clinically meaningful normative data. This information was included in participants paragraph.

Sampling Procedure: What was the attrition rate (statistical death)?

**Response:** We thank the reviewer for this question. From the original sample of 1,748 participants, 108 cases were excluded due to incomplete data, resulting in a final sample of 1,640 participants. This corresponds to an attrition rate of approximately 6.2%, which is within acceptable limits for large-scale normative studies.

Data Collection: Were questionnaires filled out on paper or online?

**Response:** We appreciate this question. All questionnaires were completed on paper, in accordance with the administration guideline of the test (see page 7).

Country-Specific Data Not Reported: The study claims that performance varies by country, but country-specific analyses are not shown in the results section.

**Response:** We thank the reviewer for this observation. In the revised manuscript, we have clarified the country-specific effects in the Results section (page 13). In addition, we improved the visualization of these effects by replotting Figure 3, and table 2.

Adding a comparison table by country (e.g., means, SDs, effect sizes) would improve clarity.

**Response:** We appreciate this suggestion. However, we decided not to include a comparison table by country based on means and standard deviations. Such tables may lead readers to interpret raw averages as normative values, which can be misleading and have been repeatedly criticized in the neuropsychological literature. Our approach, combining Item Response Theory with Linear Mixed Models, provides more robust normative estimates adjusted for relevant covariates (e.g., age, sex, parental education, country), avoiding the limitations of unadjusted descriptive statistics. To maintain clarity, we emphasize the adjusted normative data and provide a practical calculator for clinical application.

Normative Data Calculation: The example provided is useful, but the online calculator should be demonstrated visually.

**Response:** We thank the reviewer for this helpful suggestion. In the revised manuscript, we included both the link to the online calculator https://diegorivera.shinyapps.io/calculator_tamvi_tri/, where readers can obtain their own computations, similar to the next screenshot.

Normative Data Calculation: What does the calculator output look like? (e.g., a figure, table, or graph comparing the individual score to normative data).

**Response:** We thank the reviewer for this observation. In the revised manuscript, we clarified that the calculator output is presented as a table displaying the individual’s ability score, z-score, and adjusted percentile. See last comment.

Normative Data Calculation: How was the calculator validated? Are there key parameters that need to be reported for its use?

**Response:** Thank you for this important question. The online calculator was validated by cross-checking its outputs against the normative conversions obtained directly from the statistical models (IRT [supplemental S1] and LMM [table 2]). For a set of randomly selected response patterns, the calculator’s ability scores, residuals, z-scores, and adjusted percentiles were compared with the values generated by R scripts, showing perfect correspondence. Regarding key parameters, the calculator requires the following inputs: item-by-item test responses, age, sex, parental years of education, and country. The outputs provided are (a) the individual’s ability score (θ), (b) the predicted ability score based on covariates, (c) the residual and standardized residual (z-score), and (d) the adjusted percentile. These parameters are explicitly reported in the revised Normative data application section to facilitate transparency and reproducibility.

Impact of Sociodemographic Variables: The authors mention that age, country, and gender influence test performance, but the implications of these interactions are not fully discussed. How should clinicians and researchers adjust their interpretations if these factors significantly affect results?

**Response:** Given that the use of regression-based norms already takes into account the influence of these factors, such as gender, age, and country, the clinician can feel sure that their interpretations are accurate once scores are transformed from raw data to standardized units by using the calculator.

Educational System Differences: The sample includes countries with diverse education systems (low-, middle-, and high-income settings). Could differences in educational background explain some of the performance variation?

**Response:** Thank you for your comment. We agree that education systems can vary by regional income differences. Given that we entered the country as a variable in the regression model, we believe these differences are largely controlled for.

Would it be useful to control for education level as a covariate when establishing normative scores?

**Response:** We agree with the reviewer that education is a crucial variable to consider when generating normative data. In our study, we included mean parental years of education (MPE) as a covariate in the Linear Mixed Models, since the participants themselves were children and adolescents. MPE is widely used in pediatric neuropsychology as a proxy for the child’s educational and cognitive environment, and it significantly influences test performance. Importantly, we did not use the child’s own years of schooling as a covariate, because this variable is highly collinear with age in this developmental range, which could bias parameter estimation. By modeling MPE alongside age, sex, and country, our approach provides normative scores that are sensitive to educational background while avoiding multicollinearity issues. This decision is described in the Methods section (Demographic effects) and further discussed in the manuscript.

Comparison to CTT, IRT, and Rasch Modeling:

The study criticizes CTT but does not extensively discuss why IRT provides a significant advantage over traditional methods.

**Response:** We appreciate this observation. In the revised Discussion section, we expanded the comparison between CTT, and IRT

Adding a brief theoretical discussion comparing CTT, IRT, and Rasch models would strengthen the paper.

**Response:** We thank the reviewer for this suggestion. In the revised version, we added a paragraph in the Introduction section providing a concise theoretical comparison of CTT, and IRT.

The title should specify the target population (e.g., "Normative Data for Learning and Memory Test (TAMV-I) in Latin America and Spain").

**Response:** We agree with this suggestion and have modified the title accordingly. The revised title is: “Normative Data for the TAMV-I in Latin American and Spanish Children: An Item Response Theory and Linear Mixed Models Approach.” This change highlights the target population and clarifies the scope of the normative data while retaining the methodological focus of the study.

Line 90-91: There is a typo with an unnecessary period.

**Response:** We thank the reviewer for noticing this detail. The sentence was rewritten to remove the unnecessary period and to improve the flow of the paragraph.

Line 93: The author's last name should appear before the citation.

**Response:** We thank the reviewer for pointing this out. The reference has been corrected so that the author’s last name appears before the citation

Clarify Study Contribution: The introduction discusses the limitations of previous neuropsychological test standardization but does not explicitly state whether:

- Prior normative data are unreliable.
- The new approach offers an entirely different framework for interpretation.
- The study only improves the statistical methodology without changing the core interpretation of the test.

**Response:** We appreciate this insightful comment. In the revised Introduction, we clarified that prior normative data are not regarded as unreliable; however, they present two major limitations. First, existing approaches do not consider the psychometric behavior of each item in terms of difficulty and discrimination across trials, but instead rely on simple univariate analyses. Second, norms are usually calculated separately for each score, implicitly assuming independence between trials, which is not clinically accurate. Our study addresses these gaps by examining the item-level psychometric properties within each trial and by modeling them jointly using Item Response Theory combined with Linear Mixed Models. This multivariate framework yields more precise and robust normative estimates and aligns more closely with the realities of clinical assessment, where inter-trial dependencies are central to interpretation.

The authors should explicitly clarify how their results improve practical test interpretation.

**Response:** We thank the reviewer for this valuable comment. In the revised Discussion, we explicitly highlight how our results improve clinical interpretation. By incorporating item-level parameters of difficulty and discrimination across trials, and by modeling these jointly through Item Response Theory and Linear Mixed Models, the normative data provide clinicians with more precise and covariate-adjusted percentiles. Unlike traditional univariate norms that treat each trial as independent, our approach reflects the inter-trial dependencies observed in clinical practice, thereby offering a framework that is both psychometrically robust and clinically meaningful. This allows practitioners to better distinguish between normal variability and true impairment, ultimately enhancing diagnostic accuracy and treatment planning.

Figure 2: The resolution is too low, making it difficult to read.

Figure 2: Consider replotting the figure with better formatting (e.g., larger font size, clearer legends).

**Response:** We appreciate this suggestion. Figure 2 (now Figure 3) has been replotted at higher resolution, with larger font size, clearer legends, and improved formatting to enhance readability.

**Reviewer #2:**

1- The abstract lacks specificity, and there is no well-defined hypothesis or research question, making it difficult to understand the significance of the study. Without a clearly stated aim, it is challenging to assess the appropriateness of the methodology or the relevance of the findings. It should clarify the focus of the research, its importance, and the gap it intends to address or solve a problem or improve the way things are currently being done.

**Response**: We thank the reviewer for this observation. The abstract has been revised to increase specificity and clarity.

2- The writer has used certain non-mesh keywords.

**Response:** We thank the reviewer for this observation. The keywords have been revised and updated to standardized MeSH terms. The final set of keywords included in the manuscript is: Neuropsychological Tests, Memory, Child, Adolescent, Cross-Cultural Comparison, Psychometrics, Statistical Models.

3- In the final paragraph of the Results section (line 307), the symbols "@@@" are used, which are unclear. The same issue is repeated in the Discussion section at line 401.

**Response:** We thank the reviewer for noticing this error. The symbols “@@@” were unintentional and have now been replaced in the manuscript with the reference to the online calculator: <https://diegorivera.shinyapps.io/calculator_tamvi_tri/>

4- Lines 408 to 412 state that normative data cannot be generalized to an entire region due to varying influencing conditions. Doesn’t this statement itself call into question the validity of the entire study, whose primary aim was to establish normative data?

**Response:** We thank the reviewer for this observation and understand the concern. Our intention was not to suggest that the normative data generated in this study are invalid, but rather to emphasize an important clinical caution. Normative data provide robust and clinically useful references for the countries included; however, as with any large-scale normative study, they should not be assumed to account for all possible subpopulations (e.g., ethnic and racial minorities) within each country. This caveat reflects a common limitation of normative research and highlights the importance of cautious interpretation in clinical practice. We have revised the wording in the Discussion to clarify this point and to avoid misinterpretation.

5- In the Data Collection and Sampling Method section, it is unclear what measures were taken to ensure randomization. Robust sampling practices are essential for the generalization and reliability of findings, and the absence of details regarding randomization raises concerns about potential bias of the sample.

**Response:** We thank the reviewer for this important comment. The sample was not randomized but rather based on availability within the collaborating schools and universities. To minimize bias and enhance representativeness, local research teams ensured balanced distributions across age ranges and sex, and monitored mean parental education (MPE) within each subsample (see Table 1). Furthermore, the achieved sample size allowed us to estimate the expected sampling error, which confirmed that the obtained precision was within acceptable ranges for normative studies. These clarifications have been added to the Participants section.

6- Some tables are missing units of measurement, have too many decimal places, or show numbers in inconsistent formats. These small details can make the data harder to follow and take away from the overall clarity and professionalism of the presentation. The quality of visual data presentation is substandard. Figures are at times too low in resolution, poorly labeled, or lack clear scale bars, keys, and legend information necessary for independent comprehension

**Response:** We thank the reviewer for these observations. We have carefully revised all tables to ensure consistency in decimal places, and uniform formatting. In addition, figures have been replotted at higher resolution, with clearer labels and legends. All explanatory legend information is provided in the figure captions, in line with the journal’s guidelines, to facilitate independent comprehension.

7- There are no disclosures of potential conflicts of interest, funding sources, or data management protocols that would assure readers of the adherence to established ethical norms

**Response:** We appreciate the reviewer’s observation. In the revised manuscript we have included a dedicated section at the end of the paper. This section specifies the source of funding (Carolina Foundation, postdoctoral fellowship 2024), declares that the authors have no conflicts of interest, and describes the ethical approvals obtained from all participating centers.
